# Case report: An adolescent female with anosmic hypogonadotropic hypogonadism, intellectual disability, and papillary thyroid carcinoma: heterozygous deletion of *TCF12*

**DOI:** 10.3389/fendo.2024.1426916

**Published:** 2024-07-05

**Authors:** Nur Berna Celik, Abdullah Sezer, Nebiyye Genel, Senay Savas-Erdeve, İbrahim Karaman, Semra Cetinkaya

**Affiliations:** ^1^ Department of Pediatrics, Division of Pediatric Endocrinology, Health Sciences University, Dr Sami Ulus Children’s Health and Disease, Health Implementation and Research Center, Ankara, Türkiye; ^2^ Department of Genetics, Health Sciences University, Dr Sami Ulus Children’s Health and Disease, Health Implementation and Research Center, Ankara, Türkiye; ^3^ Department of Pathology, Health Sciences University, Dr Sami Ulus Children’s Health and Disease, Health Implementation and Research Center, Ankara, Türkiye; ^4^ Department of Pediatric Surgery, Health Sciences University, Dr Sami Ulus Children’s Health and Disease, Health Implementation and Research Center, Ankara, Türkiye

**Keywords:** anosmia, hypogonadotropic hypogonadism, intellectual disability, papillary thyroid carcinoma, obesity

## Abstract

**Background:**

Isolated hypogonadotropic hypogonadism is a heterogeneous clinical entity. There is a growing list of molecular defects that are associated with hypogonadotropic hypogonadism (HH). TCF12, a recently identified molecular defect, causes craniosynostosis and is suggested to be used as a biomarker for prognosis in various cancer types. Recently, TCF12 variants were shown in a cohort with HH.

**Case presentation:**

A 15.3 years old female patient was referred to the endocrinology clinic for obesity. She had been gaining weight from mid-childhood. She had her first epileptic seizure at the age of 15.1 years and mildly elevated thyroid autoantibodies were detected during evaluation for etiology of seizures. She had not experienced menarche yet. She was operated for left strabismus at the age of 7 years. School performance was poor and she was receiving special education. Tanner stage of breast was 1 and pubic hair was 3. The endocrine workup revealed hypogonadotropic hypogonadism. Also, the Sniffin’ Sticks test detected anosmia. Thyroid ultrasonography was performed due to the mildly elevated thyroid autoantibodies, and thyroid nodules with punctate calcifications were detected. Total thyroidectomy and central lymph node dissection were performed regarding the cytological findings of the nodules and multicentric papillary thyroid carcinoma with no lymph node metastasis was detected on pathology specimens. Regarding the phenotypic features of the patients, whole exome sequencing was performed and heterozygous deletion of exon 1 and exon 6–8 in *TCF12* was detected.

**Conclusion:**

Haploinsufficiency of TCF12 causes anosmic HH. Probably due to the incomplete penetrance and variable expressivity of the disease, patients could display variable phenotypic features such as intellectual disability, developmental delay, and craniosynostosis. Further description of new cases with *TCF12* variations could enhance our understanding of craniosynostosis and its potential link to Kallmann syndrome associated with this gene.

## Introduction

Isolated hypogonadotropic hypogonadism (HH) is a heterogeneous clinical entity that covers absent or incomplete puberty, constitutional delay of puberty and growth, infertility, adulthood onset hypogonadism, and hypothalamic amenorrhea (1, 2). It shows male preponderance with an incidence of 1:125.000 in females and 1:30.000 in males (2). There is a growing list of molecular defects that are associated with HH, and molecular defects could cause disordered gonadotropin-releasing hormone (GnRH) neuronal development and migration, defects in the control of GnRH secretion and GnRH action (2). Recently, Davis et al. showed a new molecular defect associated with HH and identified *TCF12* haploinsufficiency in 13 patients from 12 families with autosomal dominant/recessive Kallmann syndrome [*MIM #619718*] (3). *TCF12* (*transcription factor 12*) encodes a class-I E base-helix-loop-helix (bHLH) protein (4). It has a wide tissue expression and can form heterodimers with other bHLH proteins (4). The bHLH proteins are transcriptional regulators, involved in cell-fate determination and many processes such as neurogenesis, cardiogenesis, myogenesis, and hematopoiesis (5).

Haploinsufficiency of *TCF12* was first known to cause syndromic and non-syndromic craniosynostosis predominantly affecting coronal suture [*MIM #615314*] (4). Embryonic expression of *Tcf12* is important for the regulation of osteoprogenitors, hence suture formation (6). *TCF12* has important roles during the cortical development process for migration of the neurons (7). Patients with *TCF12* mutations display a mild behavioral and cognitive phenotype, and a slightly increased risk of social communication difficulties and psychosocial issues (8). Besides neurodevelopmental consequences associated with *TCF12* mutations, it was also suggested to be used as a biomarker for prognosis in various cancer types (9). Somatic genetic alterations of *TCF12* (i.e. overexpression in hepatocellular carcinoma, ovarian and colorectal cancer, or compromise activity in anaplastic oligodendroglioma, prostate cancer) enhance cell proliferation, migration, and invasion, are related to more aggressive cancer course (9–13). Also, Jung et al. described somatic *TCF12* mutations as a potential follicular tumor-specific thyroid mutation, which was not reported before (14).

After 13 patients with HH that were reported by Davis et al. (3), no other patient with *TCF12* mutation has been reported. Here we reported a female adolescent who presented with delayed puberty and diagnosed with HH and also papillary thyroid carcinoma during follow-up. Heterozygous deletion in *TCF12* was detected on next generation sequencing (NGS).

## Case presentation

**Figure 1:**
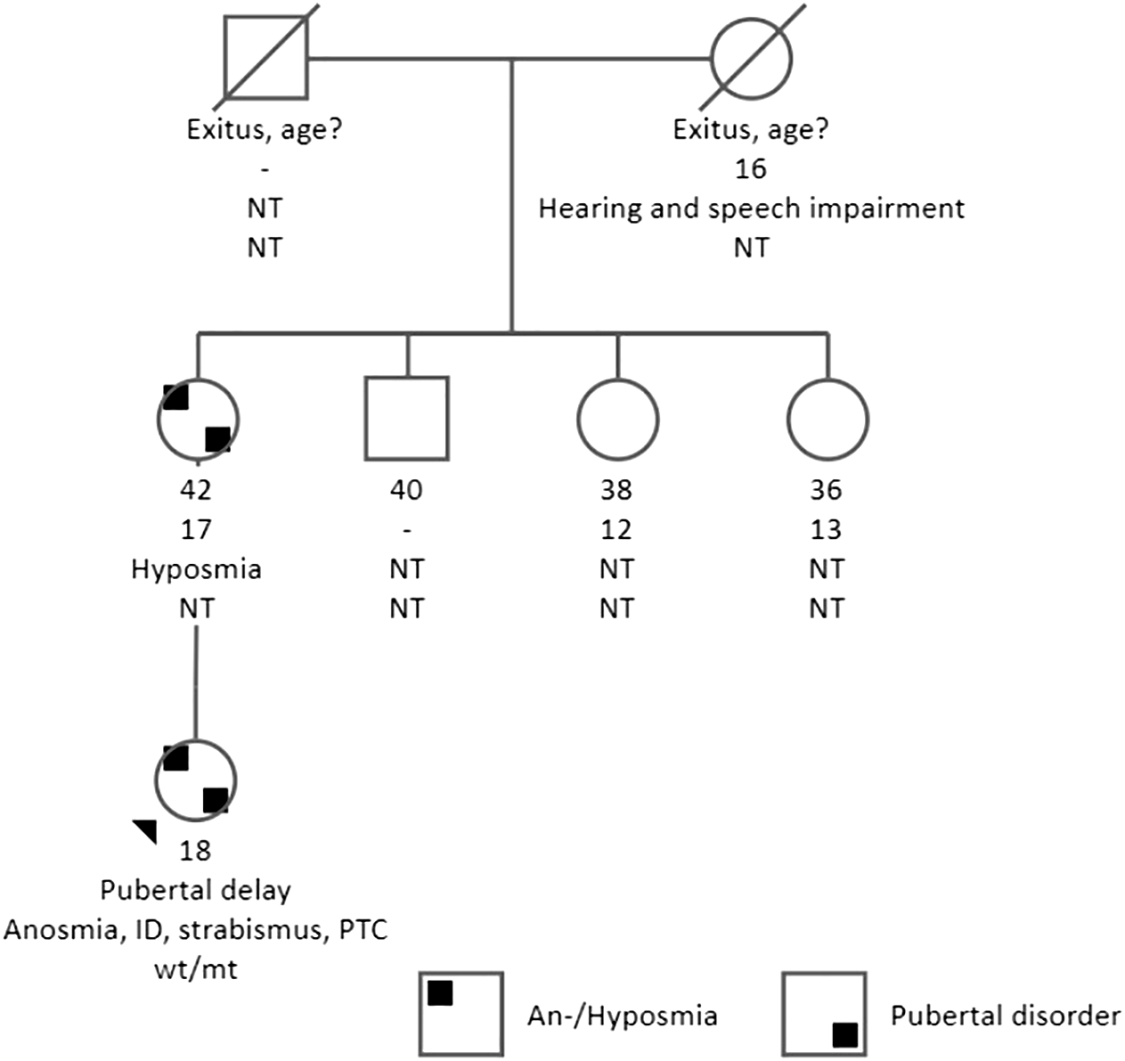
Pedigree, clinical characteristics of the family. The present age of the individuals is shown below the symbols, followed by the age at menarche, other phenotypic features, and genotype interpretation. ID, intellectual disability; NT, not tested; PTC, papillary thyroid carcinoma; genotypes are expressed by normal allele (wt) and mutated allele (mt). An arrow indicates the index case. The index case had anosmia, pubertal delay, intellectual disability, and strabismus. Also, PTC was diagnosed at the age of 16 years. Her mother had hyposmia, and the age of menarche was 16 years.

**Figure 2:**
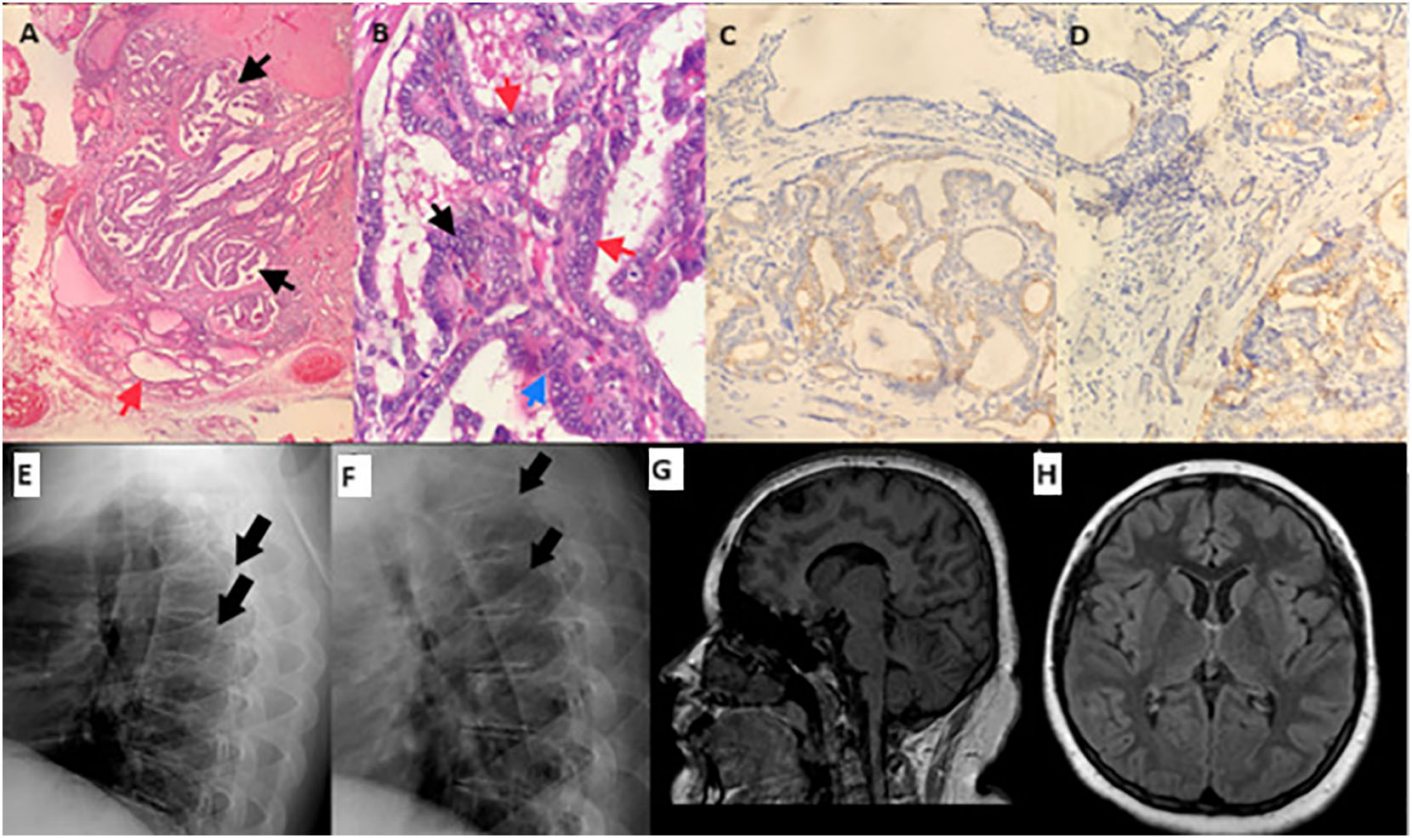
Pathology specimens, radiographs of the vertebrae, and magnetic resonance imaging of brain. Bilateral and multifocal papillary thyroid carcinoma; the largest tumor is 1.2 cm in diameter, encapsulated, and has irregular borders and infiltrative features within the gland. **(A)** A focal papillary area (black arrow), and normal thyroid tissue next to it (red arrow). **(B)** Nuclear overlapping (black arrow), intranuclear inclusions (red arrow), and nuclear grooves (blue arrow) in the tumor cells. **(C)** CK 19 immunostaining is positive in tumor cell cytoplasm. **(D)** Galectin 3 immunostaining is positive in tumor cell cytoplasm. Direct radiographies of vertebra at the time of diagnosis **(E)** and one-year after sex steroid replacement **(F)**. Mild height loss of the vertebral bodies was improved after one year of sex steroid replacement therapy. **(G, H)** sagittal and transverse sections of magnetic resonance imaging of brain display a normal skull shape.

A 15.3 years old female patient was referred to the endocrinology clinic for obesity. She had been gaining weight from mid-childhood. Her diet was high in complex and simple carbohydrates, and she was also sedentary. She did not have abnormal food-seeking behavior considering hyperphagia. She had her first epileptic seizure at the age of 15.1 years and mildly elevated thyroid autoantibodies (anti-thyroid peroxidase antibody [anti-TPO] 62.9 IU/mL [N: 0–60]) were detected during evaluation for etiology of seizures. She had not experienced menarche yet. She was born at term to healthy nonconsanguineous parents with a birth weight of 3750 grams (1.6 SDS). Developmental milestones were appropriate for her age. She was operated for left strabismus at the age of 7 years. School performance was poor and she was receiving special education. Her mother had regular menstrual periods since the age of 17 years (shown in **Figure 1**). The patient presented with a height of 0.5 SDS (165 cm), a weight of 2.5 SDS (76 kg), a body mass index of 2.1 SDS (27.9 kg/m^2^), and a head circumference of -1.2 SDS (54.5 cm) at the age of 15.3 years old. She had no dysmorphic features, thyroid gland was unpalpable. Tanner stage of breast was 1 and pubic hair was 3. Neurological examination was unremarkable except for mild intellectual disability. Total blood count, blood chemistry, and urine analysis were normal, and acute phase reactants were negative excluding metabolic, infectious, and inflammatory diseases. In endocrine workup, FSH was 0.75 mIU/mL [N: 1.8–11.5], LH <0.07 mIU/mL [N: 0.5–16], estradiol 29.4 pg/mL [N: 13–71], and insulin-like growth factor-1 125 ng/mL (<-2 SDS). Other pituitary hormones including adrenocorticotropic hormone (ACTH) (14.2 pg/mL [N: 0–46]), cortisol (9 µg/dL), prolactin (3.5 ng/mL [N: 1.9–25]), free T4 (1.2 ng/dL [N: 0.8–1.9]), thyroid stimulating hormone (TSH) (3.5 uIU/mL [N: 0.6–5.5]) were normal. Pelvic ultrasonography revealed the uterus in an infantile size (dimensions 25x14x7 mm), and ovaries in prepubertal sizes (left and right ovary was 1.8 and 0.9 mL respectively). LH-RH testing was indicative for HH (Basal and peak LH was <0.07 mIU/mL and 0.08 mIU/mL, and basal and peak FSH were <0.07 mIU/mL and 1.24 mIU/mL respectively). The Sniffin’ Sticks test detected anosmia in the patient and hyposmia in the mother. In pituitary magnetic resonance imaging, the height of the pituitary gland was 3 mm. Mild height loss was present in vertebral bodies on direct graphs (shown in **Figure 2**), and at the age of 16.5 years, estradiol was commenced at a dose of 0.5 mg/day peroral.

Thyroid ultrasonography was performed due to the mildly elevated thyroid autoantibodies, and a hypoechoic 11 mm thyroid nodule with punctate calcifications in the upper posterolateral portion, and a hypoechoic 6.2 mm thyroid nodule in the middle anterior portion of the right lobe was detected. There were no abnormal shaped and sized lymph nodes. Repeated thyroid autoantibodies were negative (anti-TPO 37.5 IU/mL [N: 0–60], anti-thyroglobulin antibody <1.3 IU/mL), and thyroglobulin was not elevated (12.2 ng/mL [N: 3.7–64]). Cytological classification of the upper posterolateral nodule was atypia of undetermined significance (Bethesda category 3), and right lobectomy with isthmusectomy was performed. Pathological evaluation revealed multifocal papillary thyroid carcinoma (shown in **Figure 2**) (the largest tumor is 1.2 cm in diameter, encapsulated, and has irregular borders and infiltrative features within the gland). Complementary thyroidectomy and prophylactic central lymph node dissection were performed thereafter. This time, papillary microcarcinoma was detected during pathologic examination and all dissected lymph nodes were negative for metastasis. I-123 whole-body scan showed remnant thyroid tissue. 100 mCi radioactive iodine ablation therapy was performed, and TSH suppressive therapy was continued thereafter.

For the molecular genetic evaluation of the patient with endocrine and neurologic problems, single-nucleotide polymorphism (SNP) microarray and whole-exome sequencing (WES) analysis were performed on the DNA obtained from peripheral blood lymphocytes, respectively. The SNP microarray study was performed with Illumina Infinium HumanCytoSNP-12 v2.1 SNP-array chips (Illumina Inc., CA) and analyzed with Bluefuse v4.5 software (Illumina Inc., CA). The sequencing library of the WES was generated using the Twist Exome 2.0 capture kit (Twist Bioscience, CA) and the sample was sequenced on the NovaSeq platform (Illumina Inc., CA). The SOPHiA DDM^®^ software and the MUSKAT™ algorithm related with it (SOPHiA Genetics, Switzerland) were used in the single-nucleotide variation (SNV) and copy-number variation (CNV) analysis of the WES data. Human GRCh37/hg19 was used as the reference genome in both analyses. Detected SNVs and CNVs are classified based on the recommendations of ACMG-AMP 2015 (15) and ACMG-ClinGen 2020 (16) guidelines, respectively.

**Figure 3:**
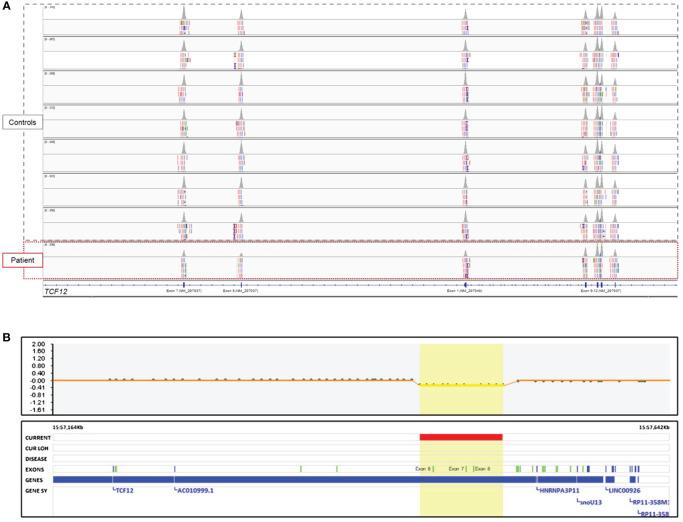
Genomic analysis of the patient. **(A)** The image showing the intragenic CNV of the *TCF12* in the BAM file of the WES data which is visualized by the Integrative Genomics Viewer (Broad Institute, MA) tool. Note that the peak volumes of the reads of the exon 7 and 8 on the NM_207037 transcript and the first exon on the NM_207040 transcript of the patient were as half as the controls studied in the same sequencing run. However, the peak volumes of the reads of exon 9–12 were similar to the controls. Deleted exon 6 could not be shown in the figure because of the distance of it to the other exons. **(B)** The image shows the intragenic CNV of the *TCF12* in the SNP microarray data.

The initial analysis of the SNP microarray with the default settings of the software did not provide any clinically significant variant. On the other hand, WES analysis revealed a CNV involving exon 1 and exon 6–8 in *TCF12* associated with HH and/or craniosynostosis (shown in **Figure 3A**), and a heterozygous missense variant, c.214C>A (p.Leu72Met) rs696217, in *GHRL* gene (NM_016362) associated with susceptibility to obesity (not shown). According to the BAM (binary-alignment map) file of the WES data, the copy-number loss variant of *TCF12* included the exon 6–8 of the canonical transcript, NM_207037, and the first exon of the NM_207040 transcript (shown in **Figure 3A**). Thereupon, the SNP microarray data was reviewed and it was noticed that the CNV in *TCF12* was missed in the first analysis (shown in **Figure 3B**). Accordingly, in the location represented by eleven probes between 57,448,455 and 57,513,293 base pairs (hg19) in the 15q21.3 chromosomal region, a heterozygous deletion with a minimum size of approximately 65 Kb and affecting the same exons as those detected in the WES analysis was found in *TCF12*. The detected CNV was not found in the healthy population database (Database of Genomic Variants, http://dgv.tcag.ca/). Heterozygous deletion of *TCF12* is classified as haploinsufficient in the ClinGen (Clinical Genomic Resource, https://www.clinicalgenome.org/) database and the haploinsufficiency of *TCF12* was associated with HH and/or craniosynostosis. Because of these properties, the CNV affecting *TCF12* was classified as pathogenic.

## Discussion

Since the first description of normosmic/anosmic HH genes in the early 1990s, the list of identified genes increased and reached more than 40 genes most of them inherited in an autosomal dominant fashion (17). Davis et al. (3) reported haploinsufficiency of *TCF12* in 13 families with anosmic HH (12 autosomal dominant, one autosomal recessive) in 2020, and also reevaluated five *TCF12* variants in a Spanish cohort with craniosynostosis and identified one with pubertal failure and anosmia (18). Here we reported an adolescent girl with a similar phenotype (HH and anosmia) who had *TCF12* deletion, and she was also diagnosed with papillary thyroid carcinoma.

*TCF12* has a wide variety of tissue expressions such as muscle, thymus, and lymphoid cells in which it has critical roles for lineage-specific gene expressions (5). *Tcf12* expression was detected in the embryonic ectoderm at high levels, in the anterior part of the subventricular zone where the olfactory neurons are generated, and along the rostral migratory pathway of the olfactory bulb (19). Suppression of *tcf12* resulted in a reduction in the length of the terminal nerve axons that provide a scaffold for migrating GnRH neurons and in the size of the olfactory bulb in a dose dependent manner (3). Also, a concomitant attenuation of other gene transcriptions that have implications in GnRH neuronal development indicates a critical role for *TCF12* in the regulation of other genes (3).

*TCF12* haploinsufficiency is a cause of syndromic and non-syndromic craniosynostosis (craniosynostosis-3, *MIM #615314*). Neurodevelopmental phenotype includes developmental delay, intellectual disability, and autism spectrum disorder with or without craniosynostosis (20). Sharma et al. (4). reported the frequency of developmental delay and intellectual disability as 13% (10/72) in their cohort of patients with craniosynostosis. Although most patients display normal developmental milestones and cognitive ability, some could still exhibit poor social, emotional, and behavioral outcomes (8). Our patient did not display craniosynostosis (shown in **Figure 2**), however, besides mild intellectual disability, she has had epileptic seizures since the age of fifteen. So far, epilepsy has been reported in only one patient who also had bicoronal craniosynostosis (8). Therefore, it is not possible to conclude epilepsy is a phenotypic feature of haploinsufficiency of *TCF12*.

Interestingly, haploinsufficiency of *TCF12* was detected both in patients with craniosynostosis and HH (3, 4). Davis et al. reported no genotype-phenotype correlation in patients with HH and/or craniosynostosis (3). The authors hypothesized that the phenotypic variability could be explained by cis or trans-acting mechanisms as well as differential mutant versus WT allelic expression throughout organogenesis during key developmental windows (3). The clinical features of our patient are similar to those of Davis et al.’s cohort (3). CNVs of *TCF12* have not been reported related to HH to date. However, some intragenic and multigenic CNVs of *TCF12* were reported in patients with craniosynostosis and/or neurodevelopmental problems (21–23). Overall, it can be said that this report, in which the intragenic deletion associated with HH is presented for the first time, will contribute to a better understanding of the TCF12-related disease group with high phenotypic variability.

In the cohort reported by Davis et al. (3) index cases and family members with the same variant exhibited one or more phenotypic features of absent puberty, learning disabilities, craniosynostosis, and anosmia indicating incomplete penetrance, variable expressivity, and pleiotropy *TCF12*-related disorders. Also, some of the patients had dental and musculoskeletal phenotypes such as hypermobility and osteoporosis (3). In our patient, mild height loss of the vertebral bodies was improved after one year of sex steroid replacement therapy, therefore osteoporosis might not be a direct consequence of *TCF12* haploinsufficiency. In that cohort, auxologic parameters had not been reported (3), however, our patient had obesity. Many of the genes associated with HH affect body mass through energy balance, appetite regulation, etc. (24). bHLH transcription factors have roles in energy balance in the hypothalamus (25), and reduced expression of *tcf12* is associated with increased fat mass (26). The age of onset of weight gain, and no food seeking behavior did not point to hypothalamic obesity actually, therefore exogenous obesity was a more likely diagnosis in our patient. However, haploinsufficiency of *TCF12* might cause less impairment of the hypothalamic regulation of body weight, and energy expenditure compared to the other genes. Also, a heterozygous missense variant in *GHRL* (p.Leu72Met) was detected in WES. Ghrelin, encoded by GHRL, is a protein predominantly secreted in the stomach. Ghrelin is the endogen ligand of the growth hormone secreting receptor (GHSR), and its stimulation causes the release of growth hormone and displays an orexigenic effect (27). Thus, it plays an important role in appetite regulation, body weight, and glucose homeostasis (28). Ghrelin levels decrease after nutrient intake and its level is inversely correlated with body mass index (29). Higher postprandial levels of ghrelin were detected in individuals with *GHRL Leu72Met* variant (30). Also, a positive correlation between the *Leu72Met* heterozygote variant and higher BMI, and early-onset obesity was detected (31). Therefore, obesity might be associated with *GHRL Leu72Met* variant in our patient.

Somatic genetic alterations of *TCF12* (overexpression or loss-of-function) could alter progression, occurrence of metastasis, and prognosis in various cancer types. It has been shown that overexpression of *TCF12* causes a more aggressive course and negatively affects prognosis by increasing cell proliferation, migration, and invasion, and inhibits apoptosis in many cancer types such as hepatocellular carcinoma, T-cell lymphoma, ovarian carcinoma, glioma, colorectal carcinoma (9, 10, 32–35). Conversely, loss-of-function of *TCF12* resulted in increased necrosis, mitotic index, cell proliferation, migration, and invasion in prostate carcinoma and oligodendroglioma (12, 13). We could not perform molecular testing or a functional study on the thyroid tissue specimen for the biological function of TCF12 in thyroid carcinoma cells. Therefore, we could not make speculations on the role of TCF12 haploinsufficiency. Papillary thyroid carcinoma is the most common thyroid cancer, and most of the genetic alterations are aggregated in the mitogen-activated protein kinase (MAPK) pathway (36). CXC motif chemokine receptor-4 (CXCR4) is the downstream target of TCF12, and its stimulation activates MAPK/ERK and PI3K/AKT signaling pathways (9). However, regarding the importance of genetic alterations on therapeutic targets as well as classification into molecular subtypes, TCF12 could be considered in future research.

In conclusion, haploinsufficiency of *TCF12* causes anosmic HH. Regarding the incomplete penetrance, variable expressivity, and pleiotropy of TCF12-related disorders, patients could display variable phenotypic features such as intellectual disability, developmental delay, and craniosynostosis. Further description of new cases with *TCF12* variations could enhance our understanding of craniosynostosis and its potential link to Kallmann syndrome associated with this gene.

## Data availability statement

The original contributions presented in the study are included in the article/supplementary materials, further inquiries can be directed to the corresponding author.

## Ethics statement

Written informed consent was obtained from the individual(s), and minor(s)’ legal guardian/next of kin, for the publication of any potentially identifiable images or data included in this article.

## Author contributions

NC: Conceptualization, Data curation, Writing – original draft, Writing – review & editing. AS: Data curation, Writing – original draft. NG: Data curation, Investigation, Writing – review & editing. SS: Writing – review & editing. İK: Data curation, Writing – review & editing. SC: Data curation, Writing – review & editing.
